# Bulletin of the Proceedings of the National Institution for the Promotion of Science

**Published:** 1841-11

**Authors:** 


ART. III.—*Bulletin of the Proceedings of the National Institution for the promotion of Science.* Established at Washington in 1840: pp. 65.

This pamphlet, No. 1 of Vol. 1, contains the proceedings of a society for the first nine months of its existence, organized in Washington City on the 15th of May, 1840. Its object is the promotion of "every branch of knowledge." Its members are divided into eight scientific classes, namely: Astronomy, Geography and Natural Philosophy; Natural History; Geology and Mineralogy; Chemistry; the Application of Science to the Useful Arts; Agriculture; American History and Antiquities; and Literature and the Arts. Two other classes should and no doubt will be established, Botany and Vegetable Physiology; and Comparative Anatomy, Physiology and Medicine.

Among the officers of this Institution we observe gentlemen of high literary and official distinction, such as the Hon. John Q. Adams, the late Gen. M'Comb, Hon. Joel R. Poinsett, Col. Joseph G. Tolten and others, of different political parties or of no political party, and others, as Dr. Thomas P. Jones, of celebrity in science.

The Institution seems to have been peculiarly active and energetic in the beginning of its labors; having held frequent meetings, and opened an extensive correspondence with men of science, and associations of the learned, both in Europe and the United States. It has also commenced the formation of a cabinet and library, intended to embrace specimens and books, illustrative of every branch of human knowledge. Among the early contributors to both, we notice the names of several distinguished physicians.

The founders of the Institution may reasonably expect two important aids from the General Government—first, the collections made by the Exploring Expedition; and second, the Smithsonian legacy. With these acquisitions, an Institution might be built up in the Capital, that would be truly and honorably national. D.

